# Elevated urinary sodium thresholds in high-salt diet heart failure: implications for diuretic resistance diagnosis

**DOI:** 10.3389/fcvm.2026.1903611

**Published:** 2026-08-13

**Authors:** Xiaotong Ji, Zheng Gao, Zhiyuan Ma, Lizhuo Li, Qingzhen Zhao, Yuzhi Zhen, Kunshen Liu, Chao Liu

**Affiliations:** 1 Heart Center, The First Hospital of Hebei Medical University, Hebei Medical University, Shijiazhuang, Hebei Province, China; 2 Department of Cardiology, Heart Center, First Affiliated Hospital of Hebei Medical University, Shijiazhuang, Hebei Province, China

**Keywords:** diuretic resistance, heart failure, high-sodium diet, prognosis, urinary sodium

## Abstract

**Background:**

Urinary sodium has been widely validated as a biomarker for diagnosing diuretic resistance (DR) in heart failure (HF) patients in Western populations; however, its role in DR Diagnosis and HF management remains unclear in high-sodium dietary regions such as northern China.

**Methods:**

In this single-center, prospective cohort study, from November 2021 to March 2024, 285 consecutive patients with acute decompensated HF (ADHF) received intravenous furosemide >80 mg/day. DR was defined as persistent fluid retention despite adequate diuretic dosing (>80 mg/day IV furosemide or equivalent), inadequate urine output (<0.5–1 mL/kg/h), or insufficient weight loss (<0.5–1 kg/day). Their urinary sodium concentrations at baseline, and at 1, 2, and 3–6 h post-administration of a 40 mg intravenous furosemide bolus were recorded.

**Results:**

In total, 177 patients were with DR and 108 non-DR. Both DR and non-DR groups exhibited markedly elevated 24-hour urinary sodium compared to western populations (130.3 in DR and 161.46 mmol/24 h in non-DR). ROC analysis indicated optimal thresholds of spot urinary sodium <85.8 mmol/L (AUC=0.791) and 1-hour post-dose urinary sodium <98.7 mmol/L (AUC=0.915) for diagnosing DR, outperforming traditional metrics. Low urinary sodium levels (spot <55.2 mmol/L, 1-hour <87.3 mmol/L) independently predicted all-cause mortality (AUC: 0.656 and 0.654, respectively).

**Conclusions:**

In high dietary sodium regions, urinary sodium thresholds for diagnosing DR are significantly higher than the western population, emphasizing the need for region-specific urinary sodium thresholds in such population.

## Introduction

Heart failure (HF) represents an advanced stage of cardiovascular disease associated with significant morbidity, poor prognosis, and considerable healthcare costs (1). Loop diuretics is a cornerstone of treatment in HF, which can effectively relieve symptoms caused by fluid overload (2, 3). However, with disease progression, many patients develop diuretic resistance (DR) (4, 5), characterized by persistent fluid retention despite high-dose diuretic therapy (typically ≥80 mg intravenous furosemide daily or equivalent), coupled with inadequate urine output (6). Recent evidence suggests that reduced urinary sodium excretion, measured via spot samples, is a sensitive and reliable marker for identifying DR and independently predicts patient mortality and hospital readmissions within six months (7–11).

Importantly, dietary sodium intake and subsequent urinary sodium excretion vary significantly across regions (12). In northern China, urinary sodium levels tend to be substantially higher compared to those observed in Western populations, largely reflecting elevated dietary salt consumption and low adherence to recommended sodium restrictions (12, 13). Such high dietary sodium intake complicates fluid management strategies and may influence diuretic responsiveness. Therefore, there is a critical need to assess the impact of high dietary sodium intake on urinary sodium-directed DR management. Our study aims to characterize urinary sodium excretion patterns in HF patients from a high-sodium dietary region, determine optimal urinary sodium thresholds for diagnosing DR, and evaluate the its prognostic role in such population.

## Methods

### Population

This single-center, prospective cohort study was registered at the Chinese Clinical Trial Registry (ChiCTR.org.cn; registration number: ChiCTR2300077973). Patients were prospectively enrolled from the inpatient cardiology service. ADHF and CHF were enrolled for analysis.

Inclusion Criteria: ADHF Group (*n* = 462): Patients with acute decompensation of chronic heart failure or end-stage heart failure, B-type natriuretic peptide (BNP) levels >100 pg/mL, and normal or reduced left ventricular ejection fraction (LVEF).

Among the 462 ADHF patients, 285 received intravenous furosemide at a daily dose of ≥80 mg and underwent diuretic responsiveness testing. The remaining 177 patients were excluded for the following reasons: 154 received lower diuretic doses (<80 mg/day intravenous furosemide or equivalent), 15 had severe renal impairment necessitating dialysis or temporary renal replacement therapy, 8 had missing urine collection data or incomplete diuretic response testing. DR was defined by persistent fluid retention characterized by inadequate urine output (less than 0.5–1 mL/kg/h) or minimal weight loss (less than 0.5–1 kg/day) despite sufficient diuretic dosing (14, 15). Of these 285 patients, 177 were classified as DR, and 108 were classified as non-DR.

Exclusion Criteria: Pregnancy or lactation; Severe renal impairment necessitating dialysis or temporary renal replacement therapy.

**Figure 1:**
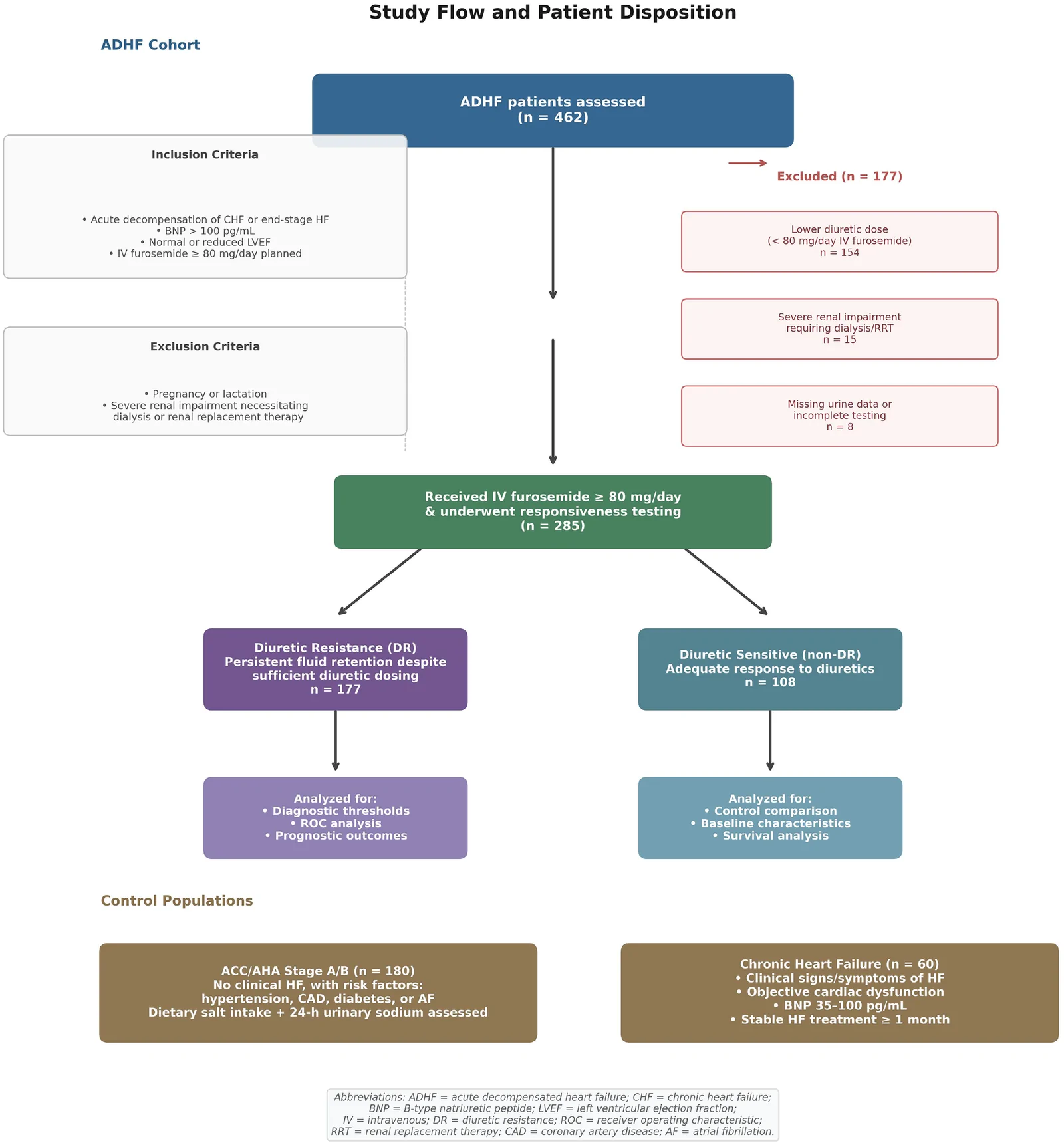
Flow chart of trial profile.

To comprehensively characterize urinary sodium excretion patterns in the general population and chronic heart failure (CHF) with high dietary salt intake in northern China, we additionally collected dietary salt intake data and corresponding 24-hour urinary sodium levels from participants classified as stage A or B (*n* = 180) according to the ACC/AHA heart failure staging system (16). These individuals had no clinical evidence of HF but presented risk factors such as hypertension, coronary artery disease, diabetes mellitus, or atrial fibrillation. This additional data allowed us to contextualize urinary sodium levels among HF patients within broader regional dietary habits. Patients with chronic heart failure (CHF) (*n* = 60). For those with CHF: participants were with clinical signs or symptoms of heart failure, objective cardiac dysfunction evidence, BNP levels ranging from 35 to 100 pg/mL, and stable heart failure treatment (including loop diuretics) for at least one month prior to enrollment (Figure 1 Flow chart of trial profile).

### Urine collection and assessment

To accurately evaluate diuretic responsiveness, a standardized diuretic response test was conducted. Patients were instructed to completely empty their bladders immediately before receiving a 40 mg intravenous bolus of furosemide. Urine was subsequently collected at precisely timed intervals: during the first hour, second hour, and third to sixth hours following 40 mg furosemide intravenous administration. At each interval, urine volume was measured, and electrolyte concentrations-including urinary sodium, potassium, and chloride - were assessed using standardized laboratory methods (Supplementary Figure 1).

For consistent interpretation, intravenous and oral furosemide dosages were standardized, where 20 mg intravenous furosemide was considered equivalent to 40 mg of oral furosemide.

### Follow-up and primary outcomes

Patients were monitored for at least six months following hospital discharge. Primary endpoints included all-cause mortality, heart transplantation, and hospitalizations attributed to heart failure.

The institutional ethics committee approved the study protocol. The study was registered at the Chinese Clinical Trial Registry (ChiCTR.org.cn; ChiCTR2300077973) and conducted in accordance with the Declaration of Helsinki guidelines.

### Statistical analysis

Data were analyzed using SPSS version 26.0 and R software. Continuous variables were presented as means ± standard deviations (SD) or medians with interquartile ranges (IQR), depending on distribution normality. Comparisons between two groups utilized Student's t-tests or Mann–Whitney U tests, whereas one-way ANOVA or Kruskal–Wallis tests were employed for multi-group comparisons. Categorical variables were analyzed using chi-square or Fisher's exact tests.

For multivariable analyses, a two-stage variable selection strategy was employed to balance statistical rigor and clinical relevance. In Stage 1, variables with statistically significant differences at baseline (*P* *<* *0.05*) were identified as candidate predictors. In Stage 2, clinically relevant variables established in the literature as important predictors of diuretic resistance—including age, left ventricular ejection fraction (LVEF), and NYHA functional class—were retained in the multivariable models regardless of univariable *P* values, to avoid residual confounding from omitting established prognostic factors. Collinearity among candidate predictors was assessed using variance inflation factor (VIF) before model construction; variables with VIF > 5 or tolerance < 0.2 were considered to have unacceptable collinearity. Specifically, eGFR and SCr exhibited high collinearity (VIF > 5); therefore, eGFR was retained in the primary model due to its clinical relevance, while SCr was excluded from multivariable analysis. All remaining candidate variables had VIF values < 3.0, indicating acceptable collinearity levels.

A backward stepwise selection approach was used for both logistic regression (DR prediction) and Cox regression (mortality prediction), with entry and retention criteria of *P* *<* *0.05* and *P* < 0.10, respectively. To address potential model overfitting, we calculated the events-per-variable (EPV) ratio for each model: the logistic regression model included 9 covariates with 177 DR events (EPV = 19.7), exceeding the recommended minimum of 10 events per variable. The Cox regression model included 6 covariates with 47 mortality events (EPV = 7.8), which is below the ideal threshold; therefore, bootstrap resampling with 1,000 iterations and 5-fold cross-validation were performed as internal validation procedures to confirm model stability and generalizability.

The final multivariable models included body mass index (BMI), systolic blood pressure (SBP), diastolic blood pressure (DBP), serum uric acid (SUA), estimated glomerular filtration rate (eGFR), B-type natriuretic peptide (BNP), right atrial diameter (RAD), and right ventricular diameter (RVD) as covariates. Logistic regression analysis identified independent risk factors for DR, and receiver operating characteristic (ROC) analysis evaluated the diagnostic performance of urinary sodium measurements at different intervals. Kaplan–Meier survival analysis compared prognoses across patient groups. Statistical significance was defined as *P* *<* *0.05*.

## Results

### Study population

Between November 2021 and March 2024, 702 participants were enrolled in the study. The median 24-hour urinary sodium excretion significantly exceeded the World Health Organization (WHO) recommended daily limit (6 g NaCl/day) in all patient groups: stage A/B (193 mmol/24 h; equivalent to 11.22 g NaCl), CHF (181.6 mmol/24 h; 10.53 g NaCl), and ADHF (178.3 mmol/24 h; 10.34 g NaCl). Spot urinary sodium levels were notably lower in ADHF patients (93.46 ± 31.43 mmol/L) compared with CHF (112.74 ± 39.34 mmol/L) and stage A/B group patients (116.5 ± 50.57 mmol/L) (*P* < 0.05), but these values remained substantially higher than those reported in Western populations (17) (Supplementary Table 1).

### Clinical characteristics

Among the 462 ADHF patients, 285 were evaluated for DR. Based on the predefined criteria for DR, 177 patients (62.1%) were categorized as DR, while 108 patients were non-DR. DR patients exhibited significantly lower 24-hour urinary sodium excretion compared to non-DR patients (130.3 mmol/24 h vs. 161.46 mmol/24 h, respectively, *P* < 0.05). Additionally, DR patients were older, had lower body weight and body mass index (BMI), and displayed lower systolic and diastolic blood pressures (all *P* < 0.05). Echocardiographic measurements indicated greater right atrial and ventricular diameters in DR patients. Laboratory assessments revealed poorer renal function in the DR group, with higher serum uric acid and creatinine levels (*P* < 0.05) (Table 1).

**Table 1:** Clinical characteristics of HF patients according to the definition of classical DR.

Variable	HF without DR group *n* = 108	HF with DR group *n* = 177	*P*
Age (y)	60.5 (47,70)	66 (53,75)	0.004
Sex (Male)	72 (66.7)	119 (67.2)	0.907
Weight (kg)	70 (68,85)	68.5 (56,76)	0.036
BMI (kg/m²)	25.95 (22.66,28.54)	24.22 (21.56,26.83)	0.022
SBP(mmHg)	125.36 ± 23.17	117 ± 24.34	0.003
DBP (mmHg)	78.5 ± 14.22	71.93 ± 12.52	0.003
Echocardiographic			
RAD (mm)	36 (32,44)	39 (32,50)	0.008
RVD (mm)	32 (30,40)	36 (31,44)	0.01
LVEDD (mm)	60 (52,69)	60 (49.5,70)	0.481
LAD (mm)	44 (39,52)	45 (38,51)	0.059
LVEF (%)	34 (24,45)	33 (24,52)	0.39
Blood data			
Fasting blood glucose (mmol/L)	5.29 (4.73,6.40)	5.83 (4.855,7.055)	0.211
Low density lipoprotein cholesterol (mmol/L)	2.59 (1.95,3.03)	2.49 (2,2.905)	0.627
Triglyceride (mmol/L)	1.17 (0.94,1.7)	1.2 (0.89,1.705)	0.402
SUA (µmol/L)	485.7 (355.35,580.3)	534.5 (431.25,687.65)	0.023
BUN (mmol/L)	7.7 (6.19,10.26)	8.17 (5.84,12.99)	0.001
SCr (µmol/L)	84.15 (69.15,104.2)	94.7 (73.75,123.3)	<0.001
Sodium (mmol/L)	139 (137,140)	138 (135,140)	<0.001
Potassium (mmol/L)	3.98 (3.74,4.3)	3.99 (3.68,4.335)	0.599
Chloride(mmol/L)	104.3 (101.3,106.1)	102.7 (99.4,105.6)	0.028
BNP (pg/mL)	773 (256,1,864)	1,075 (335,2,360.5)	<0.001
Aspartate aminotransferase (U/L)	22.6 (17.5,35.9)	23.7 (16.6,44.5)	0.967
Alanine aminotransferase (U/L)	18 (16,36.7)	24 (11,34.3)	0.027
DBIL(µmol/L)	4.2 (2.9,6.9)	5.8 (3.1,11)	0.021
IBIL(µmol/L)	14 (9.7,20.9)	15.2 (10.3,22.5)	0.463
Total bilirubin(µmol/L)	19 (12.7,25.3)	21.9 (13.6,34.4)	0.16
Glycosylated hemoglobin (%)	6.2 (5.6,7)	6.1 (5.6,6.7)	0.495
Hemoglobin (g/L)	133 ± 20.72	129.84 ± 27.93	0.12
Glomerular fltration rate (mL/min/1.73m2)	82.54 ± 30.09	68 ± 38.62	0.003
Urinalysis data			
UCr (mmol/L)	8.8 (8.8,17.7)	8.8 (4.4,8.8)	0.015
Urine microalbumin(mg/L)	30 (10,30)	10 (10,30)	0.834
Urine specific gravity	1.02 (1.015,1.03)	1.015 (1.01,1.025)	0.028
Spot urinary sodium (mmol/L)	104.29 ± 39.44	67.9 ± 31.43	<0.001
Spot urinary potassium (mmol/L)	24.57 (15.66,32.745)	21.85 (15.17,32.53)	0.618
Spot urinary chlorine (mmol/L)	101.4 (65.05,129.3)	59.65 (35.3,90)	<0.001
24-hour urinary sodium (mmol/24 h)	161.465 (101.62,265.3)	130.3 (88.45,198.9)	0.034
24-hour urinary potassium (mmol/24 h)	39.4,825 (31.40,55.11)	38 (28.11,52.1)	0.739
24-hour urinary chlorine (mmol/24 h)	148.25 (75.6,249.67)	126.3 (79.59,195.847)	0.108
24 h urine output (L)	1.8 (1.155,2.255)	1.78 (1.32,2.5)	0.539
Diuretic Response Test			
1st hour urinary sodium (mmol/L)	121.65 (114,130)	83.6 (58.4,101.25)	<0.001
1st hour urinary potassium (mmol/L)	12.905 (9.75,18.65)	18.905 (11.425,26.235)	<0.001
1st hour urinary chlorine (mmol/L)	126.4 (113.6,135.9)	89.5 (61.6,108.45)	<0.001
1st hour urine output (mL)	375 (250,500)	300 (150,400)	0.032
2nd hour urinary sodium (mmol/L)	120.3 (112.5,125.9)	89 (65.05,106.1)	<0.001
2nd hour urinary potassium (mmol/L)	12.42 (10.3,17.26)	14.64 (9.85,20.64)	<0.001
2nd hour urinary chlorine (mmol/L)	127.8 (119.6,137)	93.6 (65,115.25)	<0.001
2nd hour urine output (mL)	350 (290,500)	250 (120,400)	<0.001
3rd-6th hour urinary sodium (mmol/L)	117.2 (95,128)	79.55 (56.1,100.1)	<0.001
3rd-6th hour urinary potassium (mmol/L)	17.635 (12.98,21.75)	17.255 (13.02,23.81)	<0.001
3rd-6th hour urinary chlorine (mmol/L)	123.75 (101.1,136)	78.4 (53.45,103.55)	<0.001
3rd-6th hour urine output (mL)	475 (350,750)	40 0 (275,700)	0.268
Medications prior to admission			
Furosemide (mg)	60 (40,80)	80 (60,120)	<0.001
Spironolactone (mg)	40 (20,40)	40 (20,40)	0.042

### Diagnostic accuracy

Variables with statistically significant differences at baseline were entered into logistic regression analyses after exclusion of collinear covariates. Univariate analysis revealed that right heart enlargement, hepatic and renal dysfunction, lower BMI, higher BNP levels, and lower urinary sodium were associated with an increased risk of DR in HF patients. Multivariate logistic regression demonstrated that each 30 mmHg increment in SBP was associated with a reduced risk of DR [OR 0.559 (95% CI: 0.38–0.80), *P* *<* *0.05*]. Similarly, each 20 mmol/L increase in spot urinary sodium concentration [OR 0.179 (95% CI: 0.061–0.52), *P* *<* *0.05*], first-hour urinary sodium concentration during the diuretic responsiveness test [OR 0.028 (95% CI: 0.005–0.15), *P* *<* *0.05*], and second-hour urinary sodium concentration [OR 0.485 (95% CI: 0.127–1.846), *P* *<* *0.05*] were independently associated with a lower risk of DR. Conversely, each 500 pg/mL increase in BNP and each 5 mm increase in right atrial diameter (RAD) were associated with an increased risk of DR. Furthermore, after adjusting for BMI, SBP, diastolic blood pressure (DBP), serum uric acid (SUA), serum creatinine (SCr), estimated glomerular filtration rate (eGFR), BNP, RAD, and right ventricular diameter (RVD), spot urinary sodium and urinary sodium concentrations at 1 and 2 h during the diuretic responsiveness test remained independent predictors of DR.

**Figure 2:**
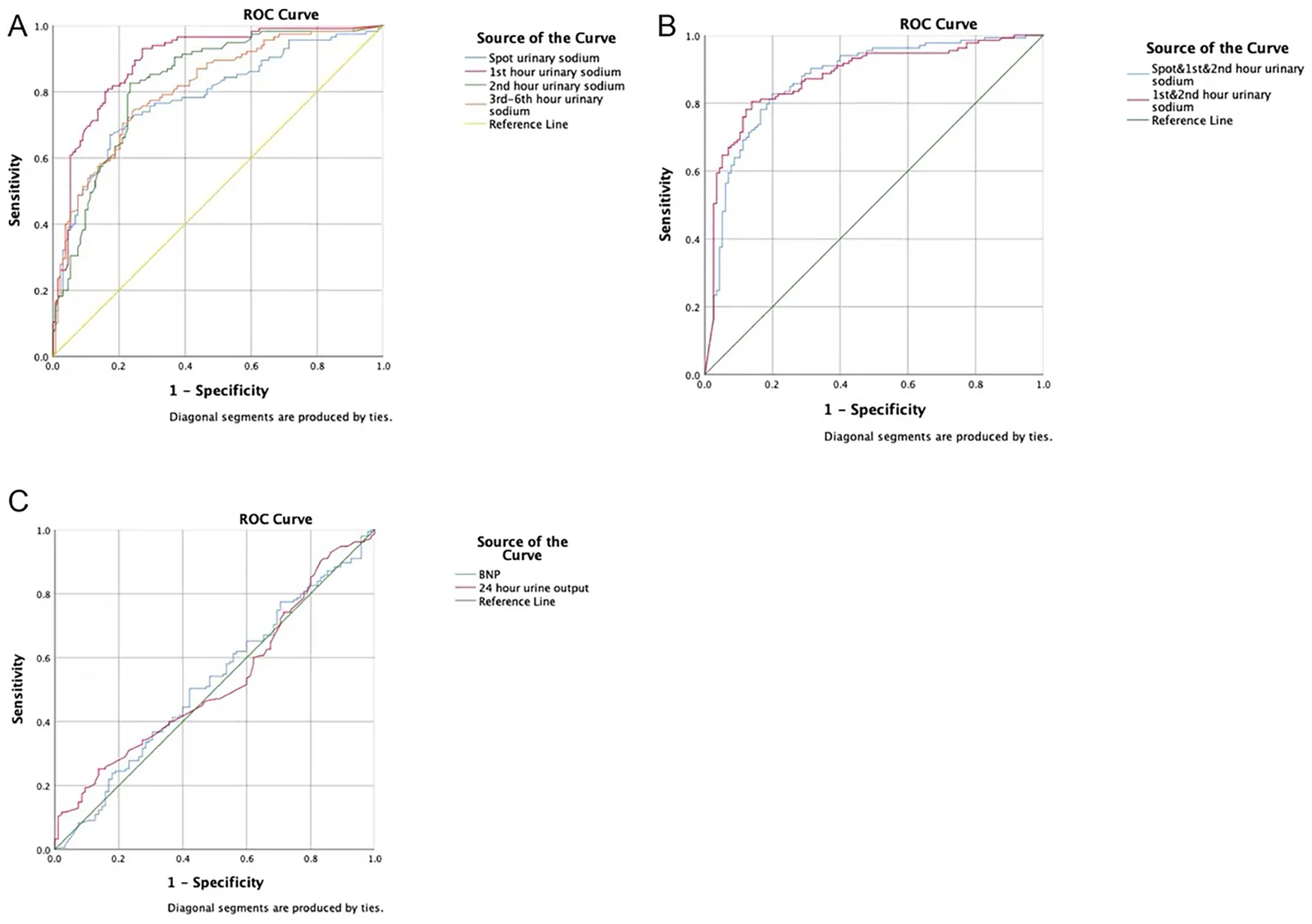
ROC curves of spot urinary sodium, 1st-hour urinary sodium, 2nd-hour urinary sodium, 3rd–6th-hour urinary sodium, BNP, and 24-hour urine output in identifying HF with DR. **(A)** ROC curve of spot urine sodium, 1-hour urine sodium, 2-hour urine sodium, 3-6 h urine sodium for single diagnosis of DR. **(B)** ROC curve of spot urine sodium, 1-hour urine sodium, 2-hour urine sodium, 3-6 h urine sodium for combined diagnosis of DR. **(C)** ROC curve of BNP and 24-hour urine volume for diagnosis of DR.

ROC analysis identified the urinary sodium concentration measured one hour after intravenous furosemide as the most accurate indicator for DR diagnosis, with an optimal cutoff of 98.7 mmol/L (AUC = 0.915, 95% CI: 0.873–0.957; sensitivity: 96.3%, specificity: 72.1%). This diagnostic accuracy exceeded spot urinary sodium levels and other collection intervals (Figure 2, Table 2). To confirm model stability and address potential overfitting, bootstrap resampling with 1,000 iterations was performed. The bootstrap-corrected AUC was 0.898 (95% CI: 0.854–0.942), closely approximating the original AUC and indicating good model stability. Additionally, 5-fold cross-validation yielded a mean AUC of 0.887 (SD: 0.031), further confirming the model was not overfitted to the derivation cohort.

**Table 2:** AUC of spot urinary sodium, 1st hour urinary sodium, 2nd hour urinary sodium, 3rd-6th hour urinary sodiumin, BNP, 24 h urine output in identifying HF with DR.

Variable	AUC	95%CI	Cut-off	*P*	Sensitivity	Specificity
Spot urinary sodium (mmol/L)	0.791	(0.721,0.861)	85.8	<0.001	0.724	0.78
1st hour urinary sodium (mmol/L)	0.915	(0.873,0.957)	98.7	<0.001	0.963	0.721
2nd hour urinary sodium (mmol/L)	0.851	(0.796,0.906)	106.8	<0.001	0.852	0.76
3rd-6th hour urinary sodium (mmol/L)	0.815	(0.752,0.877)	110.1	<0.001	0.63	0.865
1st&2nd hour urinary sodium (mmol/L)	0.922	(0.855,0.948)	–	<0.001	0.825	0.917
Spot&1st&2nd hour urinary sodium (mmol/L)	0.884	(0.833，0.935)	–	<0.001	0.832	0.911
BNP (pg/mL)	0.613	(0.595,0.811)	788	<0.001	0.689	0.519
24 h urine output (L)	0.544	(0.464，0.640)	2.25	0.252		

### Prognostic significance

**Figure 3:**
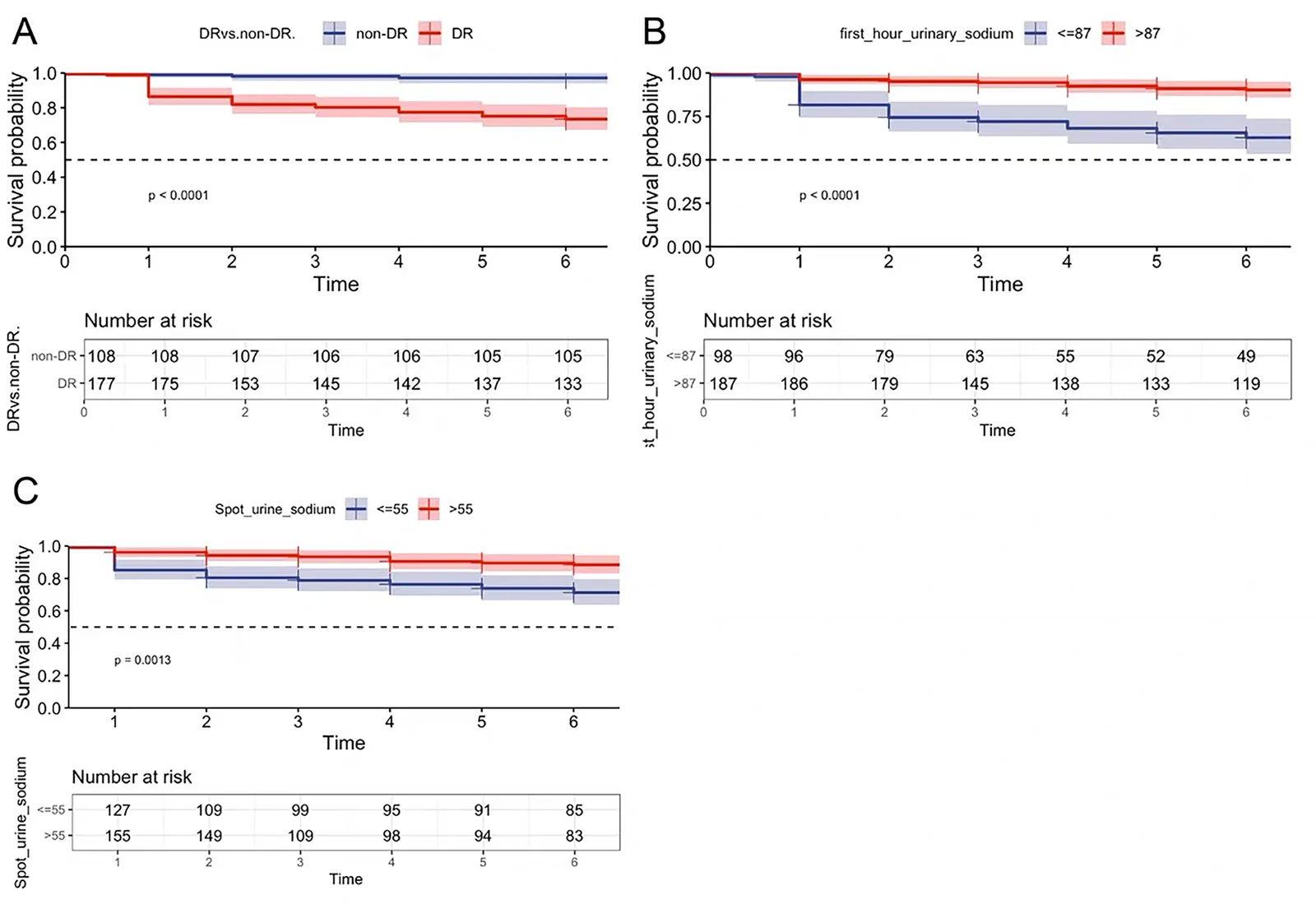
Kaplan–meier survival curves of all-cause death. **(A)** DR vs.non-DR. **(B)** 1st hour urinary sodium≤87.3 mmol/L vs. > 87.3 mmol/L. **(C)** Spot urinary sodium≤55 mmol/L vs. > 55 mmol/L.

During a median follow-up of 6 months, 47 deaths, 2 heart transplants, and 112 hospitalizations for heart failure occurred. Kaplan–Meier analysis demonstrated significantly reduced survival in patients with DR compared to those without DR (*P* *<* *0.05*). Multivariate Cox regression analysis confirmed urinary sodium levels as independent predictors of mortality, with lower urinary sodium associated with increased mortality risk (Figure 3).

## Discussion

This study systematically explored the clinical value of urinary sodium concentration as a biomarker for diagnosing diuretic resistance (DR) in heart failure (HF) patients, specifically within populations characterized by high dietary sodium intake. Our findings revealed significantly elevated urinary sodium levels among acute decompensated heart failure (ADHF) patients in northern China compared to Western cohorts. Specifically, the 24-hour urinary sodium excretion was substantially higher in both DR (130.3 mmol/24 h) and non-DR groups (161.46 mmol/24 h) compared to previously reported Western values (65 ± 45.4 mmol/24 h). This difference likely arises from regional dietary patterns involving high salt consumption, emphasizing the necessity for adjusting diagnostic thresholds for DR to reflect specific dietary and demographic contexts. Establishing spot urinary sodium <85.8 mmol/L and one-hour urinary sodium <98.7 mmol/L as diagnostic criteria for DR addresses a critical gap in HF management within this population and holds significant implications for clinical practice and public health strategies.

Our findings align with previous studies from Western populations indicating the prognostic importance of urinary sodium in patients with acute decompensated heart failure. However, we noted significantly higher urinary sodium thresholds for DR in our cohort, likely reflecting the distinct dietary habits in northern China, where salt intake is considerably greater than in Western populations (18). This regional dietary pattern underscores the importance of customizing diagnostic and therapeutic strategies for DR to local dietary practices rather than strictly applying guidelines derived from populations with different sodium consumption levels.

### Clinical value of urinary sodium as a diagnostic and prognostic biomarker

Utilizing receiver operating characteristic (ROC) analyses, we confirmed the superior performance of urinary sodium concentrations as diagnostic biomarkers for DR. Specifically, a spot urinary sodium threshold of <85.8 mmol/L (AUC=0.791) and a one-hour urinary sodium threshold of <98.7 mmol/L (AUC=0.915) provided excellent diagnostic accuracy, surpassing traditional measures such as 24-hour urine output and brain natriuretic peptide (BNP) levels. Urinary sodium measurements offer practical advantages—ease, speed, and bedside applicability—significantly enhancing clinical utility. Additionally, our study demonstrated the prognostic relevance of urinary sodium, identifying thresholds predictive of all-cause mortality (spot urinary sodium <55.2 mmol/L, AUC=0.656; one-hour urinary sodium <87.3 mmol/L, AUC=0.654). These findings underscore the dual role of urinary sodium measurements as reliable tools for early DR detection and valuable predictors for HF prognosis, ultimately guiding clinical decision-making and patient management strategies.

The pharmacokinetics of furosemide further explain the utility of the one-hour post-dose urinary sodium measurement. Since furosemide typically achieves peak plasma concentration within one hour, this interval likely provides the most accurate assessment of diuretic responsiveness (4). Patients with heart failure often experience impaired renal blood flow and increased tubular competition for furosemide transport, reducing its effectiveness despite adequate plasma concentrations—an effect that likely contributes to the reduced urinary sodium excretion observed in DR patients (6, 14, 19, 20).

### Risk factors associated with diuretic resistance

Our analyses identified several clinical factors associated with an increased risk of DR. Univariate analysis indicated that patients with DR were typically older, had lower body weight and BMI, lower blood pressure, increased right heart dimensions, worse hepatic and renal function, and a higher prevalence of NYHA class IV symptoms. Multivariate regression further demonstrated that elevated BNP levels and increased right atrial diameter (RAD) significantly correlated with a higher risk of DR, while higher systolic blood pressure and urinary sodium concentrations were associated with reduced DR risk (Table 3). These associations reflect the progressive pathophysiological deterioration typical of advanced HF, including cardiac dysfunction and systemic organ impairment. Clinically, recognizing these risk factors facilitates the identification of patients at heightened risk for DR, prompting timely adjustments in diuretic regimens and combined therapeutic approaches to mitigate adverse outcomes.

**Table 3:** Univariate and multivariate logistic regression analysis for DR diagnosis.

Variable	Univariate analysis		Multivariate analysis	
	OR(95%CI)	*P*	OR(95%CI)	*P*
BMI (kg/m²)	0.559 (0.347,0.9)	0.017	1.406 (0.418,4.73)	0.582
SBP (mmHg) per 30mmHg	0.559 (0.38,0.8)	0.002	0.13 (0.034,0.495)	0.003
DBP (mmHg) per 30mmHg	0.36 (0.19,0.66)	<0.001	3.205 (0.629,16.338)	0.161
BUN (mmol/L) per 7.1 mmol/L	1.89 (1.31,2.72)	0.01	1.282 (0.358,4.59)	0.703
SUA (µmol/L) per 200µmol/L	1.7 (1.15,2.53)	0.008	0.577 (0.198,1.682)	0.314
SCr (µmol/L)	1.69 (1.01,2.86)	0.043	2.18 (0.301,15.794)	0.441
eGFR (mL/min/1.73m2)	0.601 (0.44,0.82)	0.001	0.41 (0.114,1.47)	0.171
BNP (pg/mL) per 500pg/mL	1.14 (1.01,1.3)	0.031	1.43 (1.016,2.012)	0.04
RVD (mm) per 5mm	2.001 (1.19,3.36)	0.009	0.141 (0.02,1.006)	0.051
RAD (mm) per 5mm	1.971 (1.27,3.06)	0.003	24.804 (3.062,200.928)	0.003
Spot urinary sodium (mmol/L) per 20 mmol/L	0.25 (0.16,0.39)	<0.001	0.179 (0.061,0.52)	0.002
Diuretic Response Test				
1st hour urinary sodium (mmol/L) per 20 mmol/L	0.36 (0.18,0.79)	<0.001	0.028 (0.005,0.15)	<0.001
2nd hour urinary sodium (mmol/L) per 20 mmol/L	0.28 (0.15,0.8)	<0.001	0.485 (0.127,1.846)	0.008
3rd-6th hour urinary sodium (mmol/L) per 20 mmol/L	0.21 (0.13,0.35)	<0.001	0.407 (0.11,1.499)	0.176

The classification criteria for age, BMI, SCr, and eGFR are detailed in the Supplementary Table 2.

### Clinical utility of the diuretic responsiveness test

We introduced a practical diuretic responsiveness test based on urinary sodium measurement following a standardized intravenous administration of 40 mg furosemide. Our results showed consistently lower urinary sodium levels in DR patients across all measured intervals, with the one-hour measurement demonstrating the highest diagnostic accuracy (threshold <98.7 mmol/L; AUC=0.915; sensitivity 96.3%, specificity 72.1%). This test is simple, reproducible, and rapidly yields results, allowing clinicians to swiftly tailor individualized diuretic therapies. Compared to traditional diuretic evaluation methods, this streamlined approach enhances diagnostic efficiency and reproducibility, making it a potential standard diagnostic tool for routine clinical application.

### Prognostic significance of urinary sodium levels

During follow-up of 285 ADHF patients, we established urinary sodium concentration as an independent predictor of all-cause mortality. Cox regression and Kaplan–Meier analyses showed significantly lower survival rates among patients with reduced urinary sodium levels (spot urinary sodium <55.2 mmol/L, one-hour urinary sodium <87.3 mmol/L). Although the predictive performance of decreased urinary sodium levels for mortality was only moderate in the present study, findings from multiple international studies confirm that urinary sodium concentration serves as an important prognostic indicator in HF (7, 9, 10, 21–23). Lower urinary sodium likely signifies severe sodium retention and diminished diuretic responsiveness, highlighting the necessity for clinicians to closely monitor urinary sodium to promptly optimize treatment strategies and reduce mortality risk. This reinforces urinary sodium as an essential component of dynamic patient monitoring in HF management.

## Emerging biomarkers in diuretic resistance and heart failure risk stratification

Beyond urinary sodium, diverse biomarkers reflecting inflammation, renal tubular injury, and neurohormonal activation have been explored to refine risk stratification for DR and adverse outcomes in HF. Of particular relevance, Ömür and colleagues recently demonstrated that the neutrophil percentage-to-albumin ratio (NPAR) independently predicts both DR and mortality in HF with preserved ejection fraction, with an optimal cutoff of 13.98 (AUC = 0.892) and a robust association with all-cause mortality [HR 1.62 (95% CI: 1.31–1.94), *P* *<* *0.05*] (24). Other readily accessible inflammatory indices—including the neutrophil-to-lymphocyte ratio (25) and red cell distribution width (26)—have also been associated with HF mortality. Complementing these, specialized markers such as urinary pi-class glutathione S-transferase (pi-GST) (27), neutrophil gelatinase-associated lipocalin (NGAL) (28), and circulating cytokines (interleukin-6, interleukin-17, and soluble ST2) (29) offer mechanistic insights into tubular injury and inflammatory pathways underlying DR. Our urinary sodium findings align with this broader biomarker framework. While specialized markers provide pathophysiological insights, urinary sodium retains distinct practical advantages as a non-invasive, rapidly measurable indicator that directly reflects diuretic action. Future multi-marker strategies integrating urinary sodium with inflammatory indices such as NPAR may enable more robust risk stratification in HF management.

## Limitations

Our study has several important limitations. First, the single-center design, modest sample size, and lack of external validation limit generalizability. Second, the median 6-month follow-up may have precluded detection of longer-term prognostic associations. Third, dietary sodium was inferred from urinary excretion rather than directly assessed via standardized dietary questionnaires, introducing potential measurement variability. Fourth, the diagnostic thresholds were derived from a single-center cohort; given that urinary sodium levels are influenced by dietary habits, renal function, diuretic dosing, and local clinical practices, their generalizability remains uncertain. Fifth, although urinary sodium excretion is physiologically linked to diuretic response, its relationship with the conventional metrics defining diuretic resistance—namely urine output and weight change—is not redundant. The correlation between spot urine sodium concentration and 24-hour urine volume is modest (r = 0.25–0.52) (30), and in patients with low-to-medium urine output, spot urinary sodium content provides independent prognostic information beyond urinary volume output (31). Furthermore, urinary sodium directly quantifies natriuretic efficiency at the nephron level—a qualitative parameter that urine output and weight change cannot capture (32). Nonetheless, the exceptionally high AUC of 0.915 for 1-hour urinary sodium should be interpreted cautiously, as the metric may partially reflect this physiological interdependence. Sixth, although we performed bootstrap resampling (1,000 iterations) and 5-fold cross-validation to assess model stability, these are internal validation methods. External validation in independent, geographically diverse cohorts is essential before these thresholds can be recommended for broader clinical application. Seventh, the Cox regression model for mortality prediction had an EPV ratio of 7.8, which falls slightly below the ideal threshold of 10; although bootstrap validation confirmed the independence of 1-hour urinary sodium as a mortality predictor (bootstrap-corrected HR: 0.589, 95% CI: 0.371–0.936), the mortality prediction results should be interpreted with appropriate caution.

## Future directions

Future research should prioritize several avenues. Bootstrap or cross-validation within the current cohort, and ideally external validation in independent, geographically diverse cohorts, is essential before these thresholds can be recommended for broader clinical application. Multi-marker strategies integrating urinary sodium with inflammatory indices such as NPAR, NLR, or tubular markers such as pi-GST may achieve more robust risk stratification than any single biomarker alone. Formal comparison of the incremental predictive value of urinary sodium against conventional diuretic response parameters using net reclassification improvement and integrated discrimination improvement analyses is warranted. Additionally, prospective interventional studies examining whether region-specific, urinary sodium-guided diuretic titration improves decongestion efficiency and clinical outcomes in high-sodium dietary populations represent a critical next step.

## Conclusion

In conclusion, our study systematically evaluated urinary sodium concentration's role in diagnosing DR and predicting outcomes in HF patients from northern China. We demonstrated that region-specific thresholds may significantly enhance DR diagnosis and prognostication, suggesting urinary sodium's potential as both a biomarker and clinical management tool. Our findings provide preliminary evidence for regionally tailored HF management strategies and contribute novel insights to the broader field of HF research globally. However, these thresholds require external validation in independent, geographically diverse cohorts before they can be recommended for routine clinical implementation. Future validations and refinements may establish urinary sodium measurements and responsiveness tests as integral components of precision medicine strategies in heart failure care.

## Data Availability

The original contributions presented in the study are included in the article/Supplementary Material, further inquiries can be directed to the corresponding author.
