# Supplementary material for: Elevated urinary sodium thresholds in high-salt diet heart failure: implications for diuretic resistance diagnosis
Source: Front Cardiovasc Med. 2026 Aug 13;13:1903611. doi: 10.3389/fcvm.2026.1903611 (PMC13518530; doi:10.3389/fcvm.2026.1903611)
Supplement: Supplementary file 1 [file Datasheet1.docx]

Supplementary information

Supplementary information

Supplementary information Table 1 Clinical characteristics of study subjects (n=702)

|  | ACC/AHA Stages of HF A or B  n=180 | ACC/AHA Stages of HF C or D n=522 | | *P* |
| --- | --- | --- | --- | --- |
|  |  | CHF  n=60 | ADHF n=462 |  |
| Age（y） | 60(53,69)^*#^ | 64(51,72) | 66(53,75) | *0.004* |
| Sex（Male） | 110（61.1） | 37（61.7） | 303（65.6） | *0.483* |
| Weight（kg） | 72.25(65,82)^*^ | 70(60,80)^*^ | 70(60,80)^#^ | *0.003* |
| BMI（kg/m²） | 25.39(23.38,29.38)^*^ | 25.56(22.49,28.06)^*^ | 24.91(21.56,26.83)^#^ | *0.047* |
| SBP(mmHg) | 134±18.79^*#^ | 128.8±22.75^*^ | 121±35^#^ | *<0.001* |
| DBP(mmHg) | 83.63±11.71^*#^ | 78.8±16^*^ | 74.14±17^#^ | *<0.001* |
| Echocardiographic |  |  |  |  |
| RAD (mm) | 32(30,34)^*#^ | 34(31,36.5) ^*^ | 37(32,45) ^#^ | *<0.001* |
| RVD (mm) | 30(28.5,32)^*#^ | 31(30,34) ^*^ | 34(30,42) ^#^ | *<0.001* |
| LVEDD（mm） | 46(44,49) ^*#^ | 52(47,63) ^*^ | 60(49.5,70) ^#^ | *<0.001* |
| LAD（mm） | 34(31,36) ^*#^ | 40(36,46) | 45(38,51) | *<0.001* |
| LVEF（%） | 63(60,66.5) ^*#^ | 47(33,60) ^*^ | 33(24,52) ^#^ | *<0.001* |
| Blood data |  |  |  |  |
| Fasting blood glucose (mmol/L） | 5.04(4.64,5.93)* | 5.3(4.69,6.68) ^*^ | 5.83(4.855,7.055) ^#^ | *<0.001* |
| Low density lipoprotein cholesterol(mmol/L） | 2.57(2.08,3.00) | 2.51(1.96,3.03) | 2.49(2,2.905) | *0.544* |
| Triglyceride(mmol/L） | 1.35(1.06,1.83) | 1.26(0.9,1.77) | 1.2(0.89,1.705) | *0.036* |
| SUA(µmol/L) | 361.5(306.85,429.2) ^*#^ | 381(282.8,464.2) ^*^ | 455.4(345.2,569.8) ^#^ | *<0.001* |
| BUN (mmol/L) | 5.03(4.185,6.27) ^*#^ | 6.77(5.23,8.91) ^*^ | 8.17(5.84,12.99) ^#^ | *<0.001* |
| SCr (µmol/L) | 68(56.4,77.45) ^*#^ | 77(63.5,98.5) ^*^ | 94.7(73.75,123.3) ^#^ | *<0.001* |
| Sodium (mmol/L) | 140(139,141) ^*#^ | 139(137,141) ^*^ | 138(135,140) ^#^ | *<0.001* |
| Potassium (mmol/L) | 3.96(3.76,4.15) | 3.98(3.74,4.28) | 3.99(3.68,4.335) | *0.297* |
| Chloride(mmol/L) | 104.8(103.4,106.45)^*^ | 104.3(102.3,106.9) ^*^ | 102.7(99.4,105.6) ^#^ | *<0.001* |
| BNP (pg/mL) | 23.2(11.15,47.75) ^*#^ | 43.4（11.6，70.2）^*^ | 774.5(267,1696) ^#^ | *<0.001* |
| Hemoglobin (g/L) | 136(127,148)^*^ | 133(120,146) | 132(117.5,143.5) | *0.015* |
| eGFR (mL/min/1.73m2) | 102.64(77.8,123.9) ^*#^ | 90.23(71.62,100.21) ^*^ | 73.42(49.92,97.38) ^#^ | *<0.001* |
| Spot urinary sodium（mmol/L） | 118.54±50.57^*^ | 112.74±39.34^*^ | 93.46±31.43^#^ | *<0.001* |
| Spot urinary potassium（mmol/L） | 20.84(14.65,29.11) | 23.54(15.36,33) | 21.02(15.17,32.53) | *0.151* |
| Spot urinary chlorine（mmol/L） | 116.6(75.6,146.3)^*^ | 108.8(74.3,128.2) ^*^ | 87.65(35.3,90) ^#^ | *<0.001* |
| 24-hour urinary sodium（mmol/24h） | 193(122.5,256)^*^ | 181.6(115.2,234) ^*^ | 178.3(88.45,198.9) ^#^ | *<0.001* |
| 24-hour urinary potassium（mmol/24h） | 38.50 (26.65,45) | 38.93(25.5,49.08) | 40(28.11,52.1) | *0.334* |
| 24-hour urinary chlorine（mmol/24h） | 188.3(115.2,255.5)^*^ | 174.9(104.9,225.15) ^*^ | 126.3(79.59,195.8) ^#^ | *0.001* |
| 24 hour urine output (L) | 2.04(1.5,3)^*^ | 1.8(1.3,2.28) ^*^ | 1.78(1.32,2.5) | *0.001* |
| Medications prior to admission |  |  |  |  |
| Furosemide（mg） | 0^*#^ | 20（20，40）^*^ | 80（40，120）^#^ | *<0.001* |
| Spironolactone（mg） | 0^*#^ | 20（0，40）^*^ | 40（20，40）^#^ | *<0.001* |
| Cause of HF |  |  |  |  |
| Ischemia (%) | 128（71.1） | 34（56.7） | 236（51.1） | *<0.001* |
| IDC (%) | 0 | 16（26.7） | 175（37.9） | *<0.001* |
| Others (%) | 52（28.9） | 10（16.7） | 51（11.04） |  |
| Atrial fibrillation（%） | 13（7.2） | 10（16.7） | 158（34.2） | *<0.001* |
| Hypertension（%） | 114（63.3） | 33（66） | 187（40.5） | *<0.001* |
| Diabetes（%） | 44（24.4） | 21（35） | 157（34） | *0.098* |
| NYHA |  |  |  |  |
| I | —— | 23 | 0 |  |
| II | 0 | 37 | 0 |  |
| III | 0 | 0 | 172 |  |
| IV | 0 | 0 | 290 |  |

Supplementary information Table 2 Classification by levels

| characteristics | Classification by levels |
| --- | --- |
| BMI | 1：<18.5 kg/m²；  2：≥18.5 kg/m² and <25 kg/m²；  3：≥25 kg/m² |
| SCr | 1：<133 µmol/L；  2：≥133 µmol/L and <177 µmol/L；  3：≥177 µmol/L and <442 µmol/L；  4：≥442 µmol/L and <707 µmol/L；  5：≥707 µmol/L |
| eGFR | 1：≥90 mL/min/1.73m²；  2：≥60 and <90 mL/min/1.73m²；  3：≥30 and <60 mL/min/1.73m²；  4：≥15 and <30 mL/min/1.73m²；  5：<15 mL/min/1.73m² |

| Supplementary information Table 3 Univariate and multivariate analysis of factors for all-cause mortality in patients with HF | | | | | |
| --- | --- | --- | --- | --- | --- |
|  | Univariate analysis | | Multivariate analysis | | |
|  | HR(95%CI) | P | HR(95%CI) | P | |
| Spot urinary sodium（mmol/L）per 20mmol/L | 0.721（0.534，0.974） | 0.033 | 0.802(0.524,1.226) | 0.045 | |
| 1st hour urinary sodium（mmol/L）per 20mmol/L | 0.604（0.44，0.828） | 0.002 | 0.567(0.37,0.868) | 0.009 |  |
| 2nd hour urinary sodium（mmol/L）per 20mmol/L | 0.768（0.546，1.082） | 0.131 | 1.301(0.766,2.21) | 0.33 | |
| 3rd-6th hour urinary sodium（mmol/L）per 20mmol/L | 0.78（0，554，1.097） | 0.154 | 1(0.634,1.579) | 0.999 | |

| Supplementary information Table 4 AUC of Spot urinary sodium,1st hour urinary sodium in identifying all-cause mortality in patients with HF | | | | | | | |
| --- | --- | --- | --- | --- | --- | --- | --- |
|  | AUC | cutoff | 95%CI | P | sensitivity | | specificity |
| Spot urinary sodium（mmol/L） | 0.656 | 55.2 | (0.562,0.75) | 0.002 | 0.767 | 0.447 | |
| 1st hour urinary sodium（mmol/L） | 0.654 | 87.3 | (0.561,0.746) | 0.002 | 0.532 | 0.545 | |
